# An early prediction model for gestational diabetes mellitus based on metabolomic biomarkers

**DOI:** 10.1186/s13098-023-01098-7

**Published:** 2023-06-01

**Authors:** Melissa Razo-Azamar, Rafael Nambo-Venegas, Noemí Meraz-Cruz, Martha Guevara-Cruz, Isabel Ibarra-González, Marcela Vela-Amieva, Jaime Delgadillo-Velázquez, Xanic Caraza Santiago, Rafael Figueroa Escobar, Felipe Vadillo-Ortega, Berenice Palacios-González

**Affiliations:** 1 Unidad de Vinculación Científica, Facultad de Medicina UNAM en Instituto Nacional de Medicina Genómica (INMEGEN), Periférico Sur 4809, Tlalpan, Arenal Tepepan, 14610 Mexico City, México; 2 Laboratorio de Bioquímica de Enfermedades Crónicas Instituto Nacional de Medicina Genómica (INMEGEN), 14610 Mexico City, Mexico; 3 Departamento de Fisiología de la Nutrición, Instituto Nacional de Ciencias Médicas y Nutrición “Salvador Zubirán”, 14080 Mexico City, Mexico; 4 Instituto de Investigaciones Biomédicas, IIB-UNAM, 04510 Mexico City, México; 5 Laboratorio de Errores Innatos del Metabolismo, Instituto Nacional de Pediatría (INP), 04530 Mexico City, México; 6 Centro de Salud T-III Dr. Gabriel Garzón Cossa, Jurisdicción Sanitaria Gustavo A. Madero, SSA de la Ciudad de México, Mexico City, México; 7 Laboratorio de Envejecimiento Saludable del INMEGEN en el Centro de Investigación sobre Envejecimiento (CIE-CINVESTAV Sede Sur), 14330 Mexico City, México

**Keywords:** Gestational diabetes mellitus, Isovalerylcarnitine, Biomarkers, Tiglylcarnitine, Metabolomics

## Abstract

**Background:**

Gestational diabetes mellitus (GDM) represents the main metabolic alteration during pregnancy. The available methods for diagnosing GDM identify women when the disease is established, and pancreatic beta-cell insufficiency has occurred.The present study aimed to generate an early prediction model (under 18 weeks of gestation) to identify those women who will later be diagnosed with GDM.

**Methods:**

A cohort of 75 pregnant women was followed during gestation, of which 62 underwent normal term pregnancy and 13 were diagnosed with GDM. Targeted metabolomics was used to select serum biomarkers with predictive power to identify women who will later be diagnosed with GDM.

**Results:**

Candidate metabolites were selected to generate an early identification model employing a criterion used when performing Random Forest decision tree analysis. A model composed of two short-chain acylcarnitines was generated: isovalerylcarnitine (C5) and tiglylcarnitine (C5:1). An analysis by ROC curves was performed to determine the classification performance of the acylcarnitines identified in the study, obtaining an area under the curve (AUC) of 0.934 (0.873–0.995, 95% CI). The model correctly classified all cases with GDM, while it misclassified ten controls as in the GDM group. An analysis was also carried out to establish the concentrations of the acylcarnitines for the identification of the GDM group, obtaining concentrations of C5 in a range of 0.015–0.25 μmol/L and of C5:1 with a range of 0.015–0.19 μmol/L.

**Conclusion:**

Early pregnancy maternal metabolites can be used to screen and identify pregnant women who will later develop GDM. Regardless of their gestational body mass index, lipid metabolism is impaired even in the early stages of pregnancy in women who develop GDM.

**Supplementary Information:**

The online version contains supplementary material available at 10.1186/s13098-023-01098-7.

## Background

The American Diabetes Association formally classifies gestational diabetes mellitus (GDM) as “diabetes diagnosed in the second or third trimester of pregnancy that was not clearly overt diabetes prior to gestation” [1]. GDM affects between 2 and 38% of pregnancies worldwide. In Mexico, the prevalence has increased from 4% to over 30% [2, 3]. The prevalence varies considerably based on the diagnostic criteria and the studied sample population [4].

Multiple risk factors have been related to the development of GDM, including genetic background, age, ethnicity, excessive weight gain during the first trimester, history of GDM during previous pregnancies, familial history of GDM, tobacco use, and presence of pregestational obesity, among others [5–9]. Above all. GDM places a heavy burden on patients and is associated with higher rates of adverse neonatal outcomes such as premature delivery, shoulder dystocia, macrosomia, birth injuries, neonatal hypoglycemia, neonatal cardiac dysfunction, and stillbirth [10, 11]. In addition, several studies have demonstrated that women with GDM have seven times more risk of developing type 2 diabetes (T2D) and a higher probability of developing arterial hypertension, dyslipidemia, and metabolic syndrome than mothers without this pathology [12, 13]. Given the interaction between GDM and poor pregnancy outcomes, a greater focus is needed on preventing, screening, diagnosing, and managing GDM.

The available methods for diagnosing GDM identify women when the disease is already established, and pancreatic beta-cell insufficiency has occurred [14]. This late diagnosis exposes the pregnant woman and fetus to early and prolonged maternal hyperglycemia risks. Clinicians recognize that early diagnosis, adequate treatment, and close follow-up are essential to decreasing the incidence of diabetes complications in pregnancy and achieving a successful outcome [15]. In addition, lifestyle changes appear to be more effective the sooner they start [16]. Another recognized limitation of screening for GDM at 24 to 28 weeks of gestation is the delay in detecting GDM that develops in the first or second trimester. Besides, many may still need to be recognized and untreated without universal screening programs in Mexico. The universal 24 to 28 week oral glucose tolerance test (OGTT) limitations support the potential value of predictive early gestational biomarkers for GDM.

Metabolomics provides a practical approach that allows the simultaneous evaluation of low–molecular weight metabolites representing the metabolic statuses of cells, tissues, or organisms [17]. Also, having brought about insights into the association between metabolism and clinical conditions, metabolomics has proven to be a potential tool for assessing GDM to improve screening, monitoring, and early detection [18]. Thus far, metabolic biomarkers have been identified between the weeks when the GDM diagnosis is established [19–21] or before diagnosis [13, 22]. The biomarkers studied include fatty acids, amino acids, glucose, glycosylated hemoglobin, acylcarnitines, pregnancy-associated plasma protein-A, and others [10, 13, 18, 23, 24]. Based on the above studies, several authors have developed predictive models for the early diagnosis of GDM [16, 25–28]. However, divergences remain between the identified metabolites, the proposed models, and the studied populations.

This study’s main aim was to find serum biomarkers that allow early identification (before gestational week 18) for pregnant women who later, between weeks 24 and 28 of pregnancy, were diagnosed with GDM using the standard oral glucose tolerance tests (OGTT).

## Methods

### Study population & clinical data

This is an analytic prospective nested case–control study with clinical follow-up between 31/May/2018 and 30/June/2019. Pregnant women were randomly recruited at two primary health care units ‘‘C.S.T.II San Miguel Topilejo” and “C.S.T.III Dr. Gabriel Garzón Cossa’’ located in Mexico City, that meet the inclusion criteria which include healthy singleton pregnant women before 18 weeks gestation, age between 18 and 35 years, without any history of major medical pathologies and/or complications (T2D, hypertension, dyslipidemia, and polycystic ovary syndrome) and/or complications (abortion or premature fetal death), with a body mass index (BMI) between 18.5 and 49.9 kg/m^2^, and followed during gestation. An OGTT at 24–28 weeks of gestation was used to classify control cases that do not develop or were developing GDM. All participants signed informed consent. The study was conducted according to the guidelines of the Declaration of Helsinki and approved by Mexico’s City Ministry of Health (Registration number: 102-010-02-18).

Informed Consent Statement: Informed consent was obtained from all subjects involved in the study.

### Anthropometric and biochemical measurements

Weight, height, and samples for metabolomic studies were obtained during the first visit at early pregnancy stages (before 18 weeks of gestation) by trained personnel using standardized techniques. Venous blood samples were collected in the morning after overnight 8 h fasting and serum was kept at 2 to8 °C, centrifuged at 1500 ×*g* for 15 min, and stored at − 70 °C until their processing at the Instituto Nacional de Medicina Genómica (National Institute of Genomic Medicine, INMEGEN) in Mexico City, Mexico. Serum levels of glucose, triglycerides, and total cholesterol were assessed by an automatic chemistry analyzer (Advia 1800 Siemens, Malvern, PA, USA), while β-Hydroxybutyric acid was determined by EnzyChrom Ketone Body Assay Kit (BioAssay Systems, Hayward, CA) and insulin was determined by ELISA (Human Insulin ELISA, ALPCO, Salem, NH). All methods were standardized with internal controls and an external quality program. HOMA- IR was calculated according to the following formula: HOMA-IR = fasting insulin (µUI/mL) × fasting glucose (mmol/L)/22.5. HOMA-β was calculated according to the following formula: HOMA-β = 20 × fasting insulin (µUI/mL)/(fasting glucose (mmol/L)—3.5) [29, 30].

### Oral glucose tolerance test (OGTT)

For GDM diagnosis, a 2 h–75 g OGTT during gestational weeks 24–28 was performed according to the International Association of Diabetes and Pregnancy Study Groups (IADPSG) criteria [31]. Diagnostic values were as follows: ≥ 5.3 mmol/l (fasting blood glucose), ≥ 10.0 mmol/l (1 h) and ≥ 8.6 mmol/l (2 h). GDM was diagnosed if one or more of the diagnostic criteria values were abnormal.

### Metabolomics analysis

Concentrations of serum acylcarnitines, free carnitine, and amino acids were measured using the approach of targeted metabolomics by electrospray tandem mass spectrometry (Quattro Micro API tandem MS, Waters Inc., Milford, MA, USA). Metabolite levels in serum were analyzed using the commercial kit (NeoBase Non-derivatized MS/MS Kit, Perkin Elmer, Waltham, MA, USA). In brief, 20 μL of plasma samples were dropped onto filter paper cards (Whatman 903^™^,Schleicher and Schüell, Dassel, Germany) and dried for 4 h at room temperature in a sterile environment. The resulting spot was precisely cut off in 2 mm circles and placed into a 96-well plate, and then 190 μLof extraction solution containing a mixture of 22 stable isotope-labeled internal standards were added. The plate was sealed, incubated under stirring (30 °C at 650 ×*g* for 30 min), and then placed in a Waters autosampler. An HPLC pump (Waters 2795) was employed for the delivery of solvent, supplying a 0.1 mL/min stream of a mixture of acetonitrile:water (80:20 v:v%). Ten microliters of each sample were directly administered into the flow at 4-min intervals. A blank sample containing extraction solution and internal standards was included in each plate in triplicate, as reference. A MicromassQuattro instrument (Waters Inc., Milford, MA, USA) coupled to an ESI source in positive mode was employed. For desolvation and nebulization, nitrogen gas was utilized, while argon was employed as the collision gas [32–34].

### Statistical analysis

A descriptive quantitative statistical analysis was performed, where quantitative variables are expressed as mean and standard deviations (for parametric variables).Median and percentiles 25 and 75 were used for non-parametric variables according to their distribution. To determine the normality of the variables Kolmogorov–Smirnov tests were executed.

Differences between the study groups (control and GDM group) were evaluated through one factor multivariate Partial Least-Squares Discriminant Analysis (PLS-DA) to visualize discrimination among samples. Permutation testing was carried out to minimize the possibility that the observed separation on PLS-DA was by chance. To evaluate the association and contribution of each variable to identify the GDM group from the control group Random Forest analyses were performed following diverse selection criteria: frequency criteria and average mean criteria. Due to the nature of the study groups (no matching between them), an unconditional logistic regression analysis was performed to estimate the average effect of predictor variables (known GDM risk factors such as age, parity, and gestational BMI) on the outcome. Also, unconditional regression analysis adjusted for the covariates above analyzed was performed on the top variables selected by Random Forest analyses. Those variables with the highest ranking among the two selection criteria were selected to create a model to classify GDM cases versus controls. Using Receiver Operating Characteristic (ROC) regression curves the optimal cutting point to discriminate the study group from the controls was determined. For all statistical analyses significance was assumed when the p-value was less than 0.05. Statistical analyses were performed on: SPSS 23, RStudio 1.4.1717, Stata 15, and MetaboAnalyst 5.0 (McGill University, Toronto, ON, Canada).

## Results

### Population demographics and clinical characteristics

This study included 75 pregnant women who completed clinical follow-up; 13 were diagnosed with GDM during the OGTT test performed at 24 to 28 gestational weeks, and 62 had normal full-term pregnancies and were classified as controls. The mean age in the overall population was 27 years, the mean of gestational weeks at the first visit was 12 weeks and 5 days in the GDM group (range from 9 to 16 weeks), and 12 weeks and 7 days in the control group (range from 9 weeks and 6 days to 16 weeks and 1 day).For these cases, no significant differences between groups were found. Parity was higher in GDM group than in the control group. No significant divergences were observed in the variables of weight and height in both groups. In this study, the GDM group had a higher prevalence of grade II obesity (see Table 1).

**Table 1:** Descriptive characteristics of the study population

Variables	Control (n = 62)	Gestational diabetes mellitus (n = 13)	p value
Age (years)	25.9 (± 5.20)	28.2 (± 5.80)	0.509
Gestational age (week.day)	12.7 (± 3.10)	12.5 (± 3.50)	0.306
GDM prevalence	–	17.3%	–
Parity	1 (1–1)	3 (2–4)**	**0.0001**
Weight (kg)	66.0 (± 13.8)	72.0 (± 19.8)	0.081
Height (m)	1.57 (± 0.06)	1.53 (± 0.07)	0.567
Gestational BMI (kg/m^2^)	26.6 (± 5.16)	30.4 (± 6.79)	0.159
Normal weight (%)	28 (45.1)	3 (23.0)	1.000
Overweight (%)	19 (30.6)	2 (15.3)	1.000
Obesity I (%)	11 (17.7)	3 (23.0)	0.356
Obesity II (%)	3 (4.83)	4 (30.7)	**0.013**^**a**^**, 0.021**^**b**^
Obesity III (%)	1 (1.61)	1 (7.60)	0.231

Bold values denote statistical significance at the p < 0.05 level

Results are shown as mean (± S.D.) for parametric variables and as median (p25-75) for non-parametric variables **p < 0.0001, Mann Whitney U test for independent samples

^a^vs normal weight BMI

^b^vs overweight BMI; Fisher’s exact test

No significant differences in basal concentration of glucose, cholesterol, β-hydroxybutyrate, and insulin were seen between groups (see Table 2). Women that later developed GDM had significantly higher triglycerides concentration compared to the control group. However, both groups are within the ranges found during the first trimester of pregnancy (1.95–2.21 mmol/L) [35]. Marginal significant differences were found in the HOMA-IR index and HOMA-β between groups, where the group with GDM presented a lower HOMA-IR index and HOMA-β compared to the control group.

**Table 2:** Biochemical parameters of the study population

Variables	Control (n = 62)	Gestational diabetes mellitus (n = 13)	p value
Glucose (mmol/L)	4.52 (± 0.56)	4.47 (± 0.55)	0.660
Triglycerides (mmol/L)	1.46 (± 0.44)	1.88 (± 0.74)*	**0.025**
Total cholesterol (mmol/L)	4.51 (± 0.80)	4.60 (± 0.56)	0.477
β-hydroxybutiric acid (μmol/L)	42.4 (± 26.4)	48.9 (± 25.0)	0.376
Insulin (pmol/L)	53.4 (± 38.1)	34.7 (± 22.5)	0.213
HOMA-IR	1.47 (± 1.02)	0.91 (± 0.51)*	**0.049**
HOMA-β	129 (78.5–196)	56.7 (29.9–196)^+^	**0.041**

Bold values denote statistical significance at the p < 0.05 level

Results are shown as mean (± S.D.) for parametric variables and as median (p25-75) for non-parametric variables. *p < 0.05, StudenT-test for independent samples. ^+^p < 0.05, Mann Whitney U test for independent samples

### Acylcarnitines and amino acid profile in the early stages of pregnancy

The acylcarnitine concentrations are described in Additional file 1: Figure S1. Significant differences were found in all the acylcarnitine species evaluated except for free carnitine (C0) and butylcarnitine (C4). About short chain acylcarnitines, the concentration of acetylcarnitine (C2) decreased and the concentrations of propionylcarnitine (C3), butylcarnitine (C4), isovalerylcarnitine (C5) and tiglylcarnitine (C5:1) increased in the GDM group. In the group of medium chain acylcarnitines, significant differences were found in hexanoylcarnitine (C6), octanoylcarnitine (C8), octenoylcarnitine (C8:1), decanoylcarnitine (C10), decenoylcarnitine (C10:1), dodecanoylcarnitine (C12) where the concentrations of these acylcarnitines were higher in the GDM group compared to the control group. Lastly, the concentrations of long chain acylcarnitines (myristoylcarnitine (C14), tetradecenoylcarnitine (C14:1), tetradecadienoylcarnitine (C14:2), palmitoylcarnitine (C16), hexadecenoylcarnitine (C16:1), stearoylcarnitine) (C16:1), stearoylcarnitine (C16:1) (C16:1), stearoylcarnitine: 1) and linoleylcarnitine (C18:2)) were higher in the GDM group compared to the control group. Regards amino acid concentrations (Additional file 2: Figure S2), the group with GDM had higher concentration of glycine, alanine, valine, citrulline, and proline compared to the control group; while the concentrations of arginine, ornithine and methionine were lower in this group compared to the control group. No significant differences were observed between the groups in leucine, phenylalanine, and tyrosine concentrations.

### Serum early pregnancy metabolite profile

**Fig. 1:**
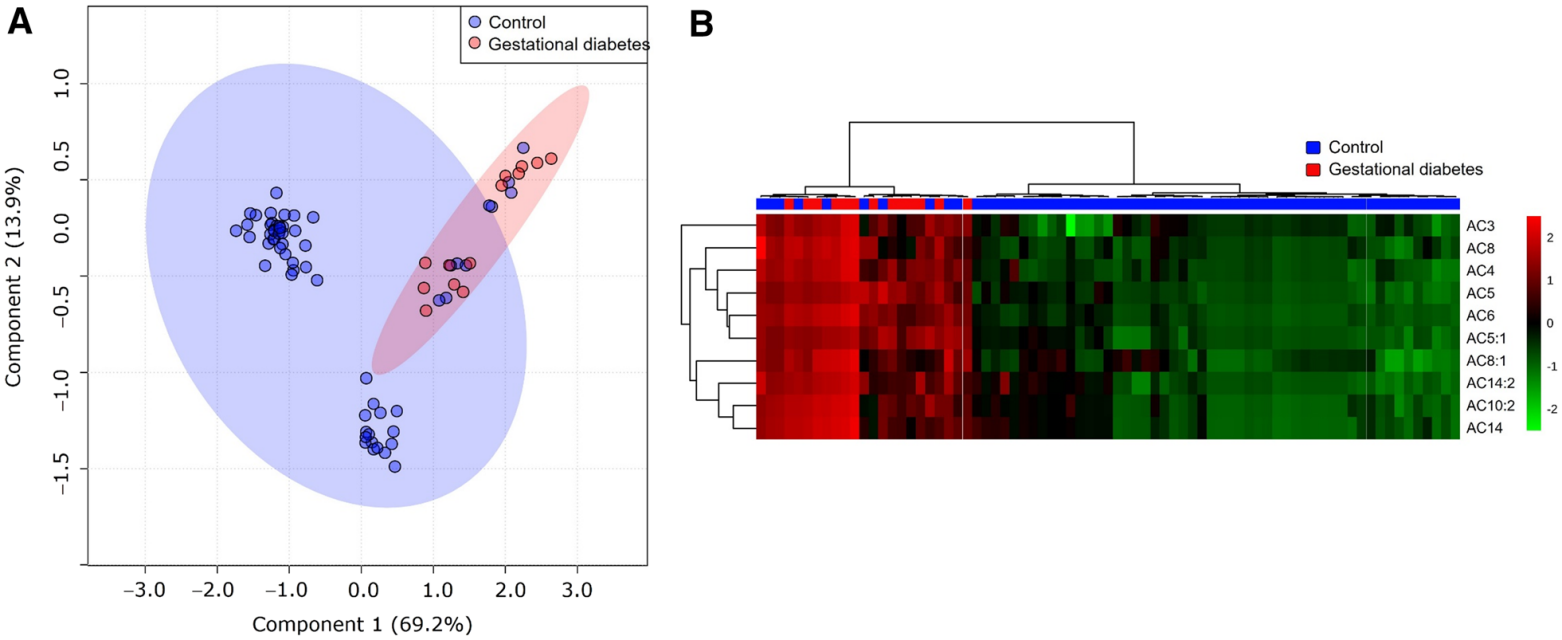
Serum metabolite profile in early pregnancy. **A** PLS-DA plot shows separation between groups; control (blue circles) and gestational diabetes mellitus (red circles). The explained variances are shown in brackets (PC1 (69.2%) and PC2 (13.9%; accuracy: 0.83, R2 = 0.42, and Q2 = 0.28: permutation p-value = 0.13); **B** Hierarchical heatmap, red and green, indicate increase and decreased concentration, respectively

To visualize the differences among the metabolites data, this research performed PLS-DA (see Fig. 1A). The score plot revealed differences in groups, which were well separated Principal Component 1 (PC1) (69.2%) and Principal Component 2 (PC2) (13.9%). The cumulative contribution rate reached 83.1%, which means that the selection of two principal components can explain the data variability among groups. The performance scores of the PLS-DA analysis for the study groups were accuracy = 0.83, R2 = 0.42, and Q2 = 0.28. Furthermore, unsupervised hierarchical clustering of abundance heatmap showed a separation between the groups (see Fig. 1B). A distinctive pattern dependent on GDM was observed. Briefly, the GDM group showed a higher concentration of propionylcarnitine (C3), octanoylcarnitine (C8), butylcarnitine (C4), isovalerylcarnitine (C5), hexanoylcarnitine (C6), tiglylcarnitine (C5:1), octenoylcarnitine (C8:1), decadienoylcarnitine (C10:2), tetradecadienoylcarnitine (C14:2), and tetradecanoylcarnitine (C14).

### Predictive accuracy through random forest analysis

**Fig. 2:**
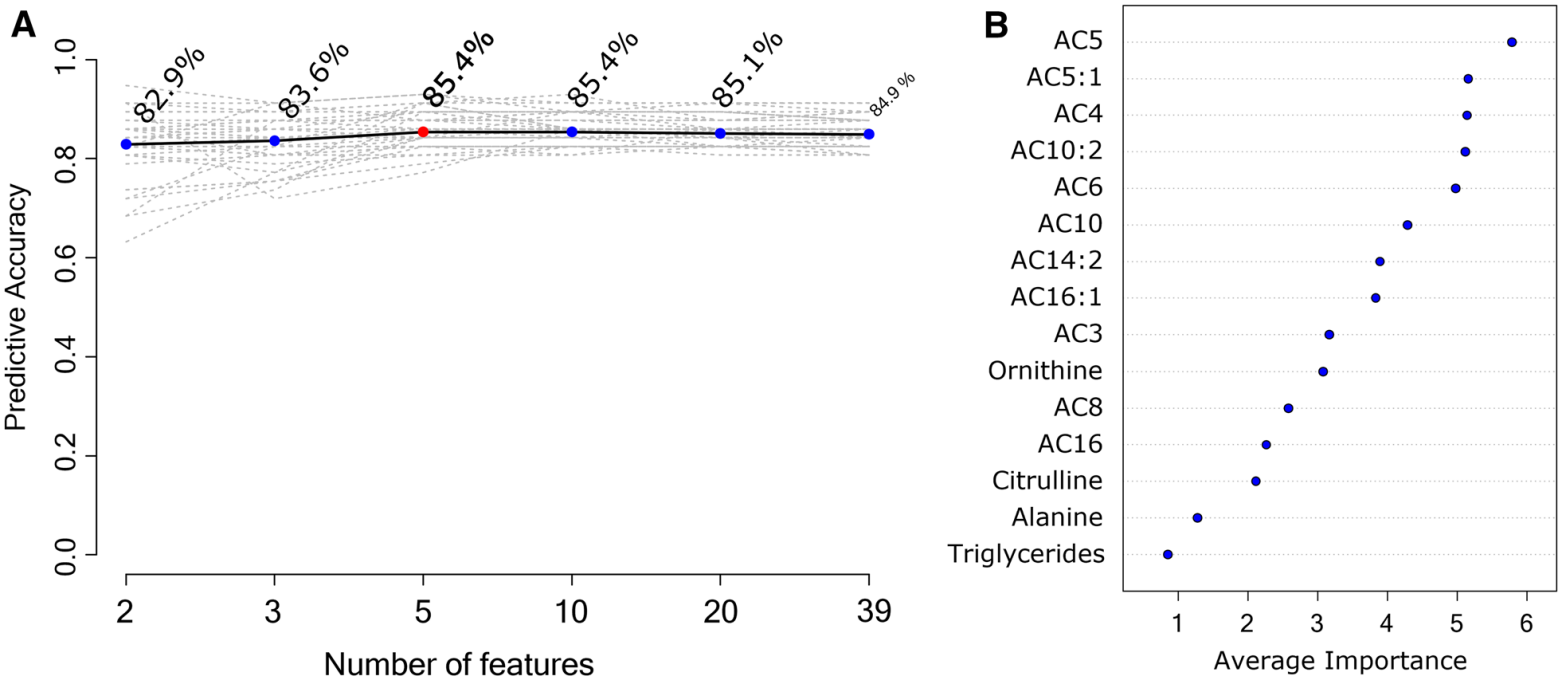
Metabolite selection through Random Forest analysis. **A** Predictive accuracy graph through Random Forest analysis. The best model that fits the highest predictive accuracy range is a model that combines 10 variables with a predictive accuracy of 85.4% (red circle). **B** The top 15 metabolites with the highest average importance to identify GDM cases from controls

Despite the lower R2 and Q2 observed in PLS-DA, an alternative to evaluating the relationship between metabolomic variables and the development of GDM is by using Random Forest decision tree analysis and thus obtaining different discrimination models. Figure 2A shows the predictive accuracy of the study´s model according to the number of features important to discriminate groups. Using a prediction model composed of only two variables (due to the small sample size) provides a prediction accuracy of 82.9% to discriminate the GDM group from the control group (see Fig. 2A). To select the candidate metabolites, the criteria of medium importance were used. Figure 2B shows isovalerylcarnitine (C5) and tiglylcarnitine (C5:1) as the metabolites with the highest average importance for discriminating the GDM group. These metabolites were adjusted for covariates (age, parity, and gestational BMI) (see Table 3).

**Table 3:** Unconditional logistic regression analysis of GDM outcome according to covariates and serum concentrations of selected ACs (isovalerylcarnitine (C5) and tiglylcarnitine (C5: 1)) adjusted by covariates (age, parity, and gestational BMI)

Characteristic	β coefficient	Standard error	Wald test value	Significance	95% CI
Age per 1 year increment	0.0817	0.0586	1.40	0.163	− 0.0330–0.1966
Parity per 1 pregnancy increment	1.2923	0.3457	3.74	**0.0001**	0.6147–1.9700
Gestational BMI per 1 unit kg/m^2^ increment	0.1190	0.0559	2.13	**0.033**	0.0093–0.2287
Age + parity + gestational BMI per 1 unit increment	0.0012	0.0003	3.77	**0.0001**	0.0006–0.0019
*Adjusted by age, parity, gestational BMI*	β coefficient	Standard error	Wald test value	Significance	95% CI
C5 (isovalerylcarnitine)per 1 µmol/L increment	24.8118	5.7870	4.29	**0.0001**	13.4693–36.1543
C5:1 (tiglylcarnitine) per 1 µmol/L increment	24.9211	5.8966	4.23	**0.0001**	13.3639–36.4783
C5 + C5:1 per 1 µmol/L increment	92.8482	25.5747	3.94	**0.0001**	46.6426–139.053

Bold values denote statistical significance at the p < 0.05 level

Estimated βcoefficient with Wald 95% confidence interval in covariates alone and after adjustment for covariates (age, parity, and gestational BMI) are shown for the selected metabolites previously on the Random Forest analysis (standardized serum acylcarnitines concentrations)

An analysis was also carried out to establish the concentrations of the acylcarnitines that make up the model for the identification of the GDM group, obtaining concentrations of isovalerylcarnitine in a range of 0.015–0.25 μmol/L and of tiglylcarnitine with a range of 0.015–0.19 μmol/L.

**Fig. 3:**
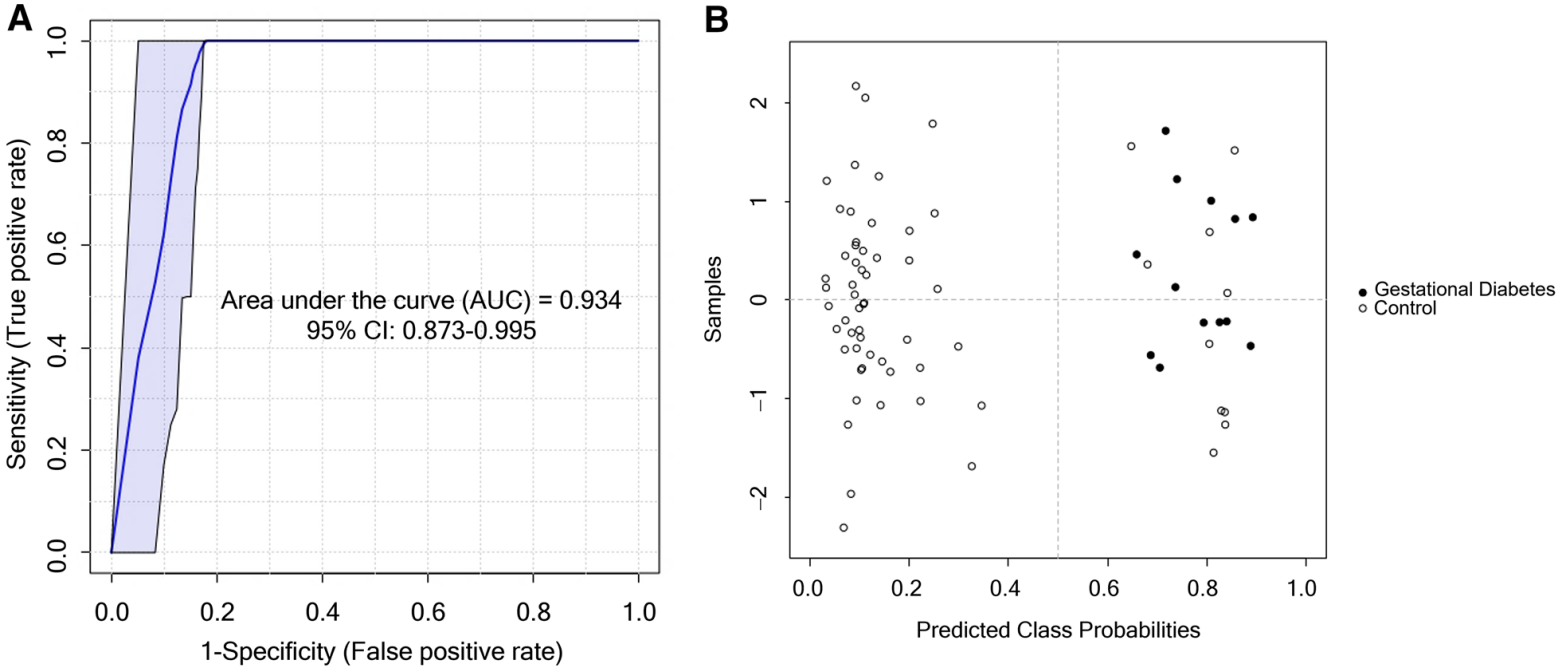
ROC curve of model for the early identification (< 18 gestational weeks) of gestational diabetes mellitus. **A** ROC curve adjusted by age, parity, and BMI, AUC = 0.934 (0.873–0.995, IC 95%) **B** Predicted class probabilities for all samples (controls (open circle) and gestational diabetes mellitus group (filled circle)) using the created biomarker model

An analysis by Receiver Operating Characteristic (ROC) curves was performed to determine the classification performance of the acylcarnitines identified in the study, obtaining an Area Under the Curve (AUC) of 0.934 (0.873–0.995, 95% CI) (see Fig. 3A). Finally, the contingency table of the corresponding model was made to evaluate classified and misclassified cases of both study groups (see Fig. 3B). The model correctly classified all cases with GDM, while it misclassified ten controls as in the GDM group.

**Fig. 4:**
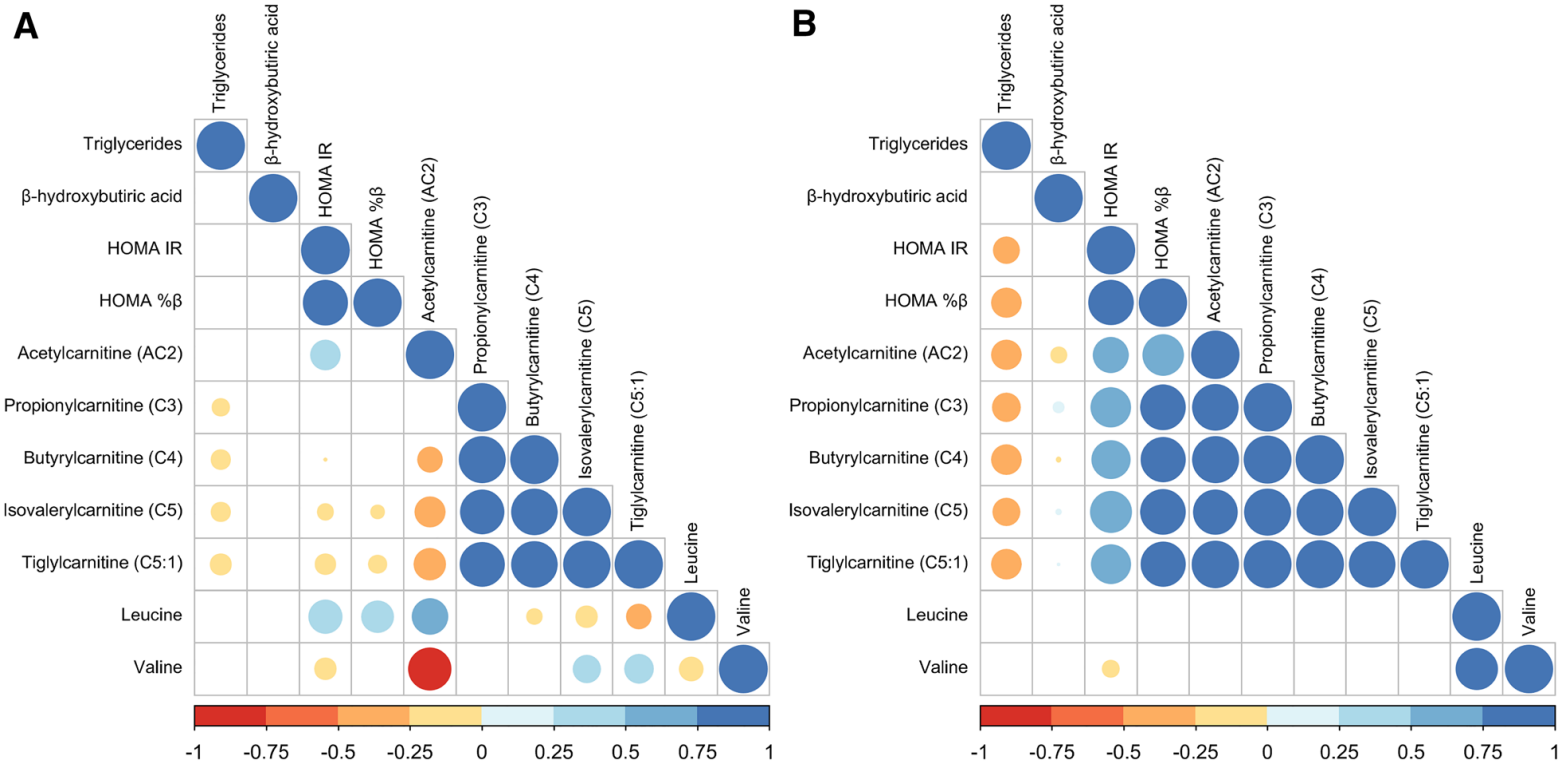
Correlation heatmap between metabolites in the early stages of pregnancy. **A** Control group. **B** Gestational diabetes mellitus group. Blue and red colors indicate an increase and de-creased correlation, respectively. The size of each dot was associated with the p-value, where a big circle represents a smaller p-value

To evaluate the association between short-chain acylcarnitines, branched-chain amino acids (BCAAs), insulin sensitivity, triglycerides, and β-hydroxybutyric acid, a correlation analysis was performed (see Fig. 4A–B). HOMA-IR and HOMA-β positively correlated with short chain acylcarnitine concentrations in the GDM group. In contrast, in the control group, a negative correlation was observed between these acylcarnitines with HOMA-IR and HOMA-β, except for acetyl carnitine. Triglycerides negatively correlate with short-chain acylcarnitines, HOMA-IR and HOMA-β. Regarding β-hydroxybutyric acid, significant correlations (negative: AC2 and AC4; positive: AC3, AC5, and AC5: 1) were observed exclusively in GDM group. Finally, leucine correlated positively with HOMA-IR and HOMA-β, only in the control group, while valine negatively correlated with HOMA-IR in both groups.

## Discussion

This study generated a metabolomic model distinguishing women who later develop GDM during early pregnancy. Short chain acylcarnitines (isovalerylcarnitine C5 and tiglylcarnitine C5:1) during weeks 12 to 18 of pregnancy allow the identification of women who will develop GDM. Incorporating biomarkers that precede the onset of hyperglycemia into a risk prediction model for GDM may facilitate earlier risk assessment, screening, and diagnosis, thereby reducing the risk of adverse maternal and infant outcomes through targeted intervention.

Several metabolomic models have been developed in the Caucasian population to diagnose GDM early. However, the predictive performance of these models varies between 0.741 and 0.848 [25–27]. This research proposed model showed a predictive performance of 0.93 (0.869–0.991, 95% CI), which allows for classifying cases of GDM correctly. This is the first study to evaluate the metabolomic profiles of pregnant Mexican women who will develop GDM before their 18-week gestation. Nevertheless, the results must be considered cautiously due to the study's limited sample size.

Some acylcarnitines species, amino acids, and β-hydroxybutyric acid evaluated in this study have been associated with obesity, insulin resistance, T2D, and GDM [12, 26, 36–39]. In T2D, branched-chain amino acids, such as leucine, valine, and isoleucine, and their intermediary metabolic products, such as short-chain acylcarnitines (C3, C5), propionyl-CoA, and acetoacetyl-CoA, have been proposed as potential biomarkers that help explain diabetic stages' pathophysiology [40]. Regarding GDM, the associations drawn between some acylcarnitines and amino acids have identified potential biomarkers of this pathology. Roy et al. noted acetylcarnitine, butylcarnitine, isobutyrylcarnitine, glutamic acid, and leucine as potential biomarkers [26]. Meanwhile, the Nevalainen et al. early pregnancy study found 3-hydroxy-isovalerylcarnitine, arginine, and glycine to be differentially expressed in women who later develop GDM [16]. Additionally, Batchuluun et al. discovered that women with GDM had higher levels of medium-chain acylcarnitines (namely C6 and C8), which are key in the progression to type 2 in women with a history of GDM [41]; furthermore, these metabolites have the potential to induce pancreatic β-cell dysfunction. In particular, the short-chain acylcarnitines used to generate the early identification metabolomic model are metabolites from branched-chain amino acid (BCAA) metabolism. Isovalerylcarnitine is a metabolite from leucine and isoleucine metabolism (through the action of isovaleryl-CoA dehydrogenase), and tiglylcarnitine or 3-methyl-crotonyl carnitine is a metabolite from isoleucine metabolism (through action acetoacetyl-CoA thiolase). Interestingly, β-hydroxybutyric acid concentrations were positively correlated with AC5 and AC5:1. It is important to note that the correlation of β-hydroxybutyric acid with short-chain acylcarnitines was present only in the GDM group.

Isovalerylcarnitine is elevated in obesity (body fat and waist-to-hip ratio), liver fat, and cardiovascular diseases [22, 42]. Serum accumulation of acylcarnitines in obese and type 2 diabetic individuals has been associated with incomplete fatty acid (FA) oxidation. The proposed mechanism is associated with an increase in β-oxidation; it causes acetyl-CoA accumulation, which exceeds the tricarboxylic acid (TCA) cycle rate, leading to an incomplete β-oxidation. Otherwise, Sunny et al. indicated that insulin stimulation resulted in higher oxidation rates of branched-chain amino acids (BCAAs), contributing to higher levels of isovalerylcarnitine in plasma [43]. The increased concentrations of BCAAs overload the catabolic pathways in the liver and skeletal muscle, increasing the production of the catabolites succinyl-CoA and propionyl-CoA and reducing the β-oxidation of fatty acids and the catabolism of glucose. Therefore, the loss of efficiency in oxidative metabolic pathways amplifies the oxidation of partially oxidized products, increasing mitochondrial stress, reducing insulin sensitivity, and altering circulating glucose concentrations [44].

In a prospective observational cohort study, propionyl‐ and isovalerylcarnitine concentrations were positively correlated with triglycerides, C‐peptide, insulin, β‐cell activity, and insulin resistance [45]. These findings are consistent with this study, in which the concentrations of isovalerylcarnitine, tiglylcarnitine and propionylcarnitine were positively correlated with insulin sensitivity and β-cell functionality. However, an interesting finding is that this correlation could only be observed in women who later developed GDM. Among the major physiological adaptations during pregnancy are insulin sensitivity, pancreatic β-cell hypertrophy, and hypersecretion [46]. Insulin secretion rises progressively throughout gestation, reaching its highest point during the third trimester [47]. In the present study, the GDM group was characterized by normoglycemia and a lower HOMA-IR index than the control group, suggesting the group that develops GDM has greater sensitivity to insulin than the control group in the first trimester of pregnancy. Remarkably, when the β-cell functionality was estimated, it was observed that women who develop GDM have a lower HOMA-β index during early gestation. This index has a correlation coefficient of 0.87 with the hyperglycemic clamp and continuous glucose in-fusion with model assessment (CIGMA) [30]. However, the HOMA-β index has not been well studied in GDM. Endo et al. showed that women who developed GDM later in pregnancy had lower HOMA-IR and HOMA-β in the first trimester of pregnancy than controls [48]. The study findings agree with Endo et al. and suggest that, unlike normal pregnancy, where greater β-cell functionality is observed, as gestation progresses, β-cell dysfunction occurs in women who develop GDM against a background of chronic insulin resistance. Therefore, insulin secretion in women who develop GDM can increase by advanced gestation; however, the insulin secretion rate in women with GDM from early gestation is lower than in healthy women.

Scott et al., reported lower glycine concentrations in women with GDM [49]. This study differs from these observations. The difference may be related to the week of gestation they were studied (14 to 27 weeks of gestation vs. 13 weeks of gestation in this study) and the matrix assessed (urine vs. serum in this study). This research strongly underlines that consideration should be taken when comparing studies using various biofluid matrices since metabolite expression can be affected by factors like ethnicity, age, circadian rhythms, diet, physical activity, and environment as pre-analytical processing [50, 51]. Notably, the alanine level is positively associated with insulin resistance and the risk of T2D [52]. Glycine and alanine are gluconeogenic amino acids. Dimou et al., indicates glycine is converted to glucose during pregnancy due to pyruvate dehydrogenase inhibition [53]. These findings suggest that during the first trimester of pregnancy, the fetoplacental unit in women who later develop GDM would be exposed to higher gluconeogenic substrates and/or glucose concentrations. A previous report indicates that as pregnancy progresses, women who develop GDM have lower ornithine concentrations [54], which was confirmed in this study. Recently, it was shown that subjects with T2D have lower serum concentrations of ornithine. The authors note that these concentrations could be attributed to the increased expression of ornithine decarboxylase observed in the early stages of diabetes [55].

Glucose is the main energetic substrate; however, when glucose is not available as an energetic substrate in insulin resistance states, the cell uses free fatty acids, lipids, and amino acids as alternative substrates, causing an imbalance between acylcarnitines and amino acids [56]. This metabolic shift causes the accumulation of intermediary substrates, such as acylcarnitines that could be implied to interfere with insulin sensitivity, causing insulin resistance and the onset of diabetes. These findings suggest that lipid and fatty acid metabolism could constitute an early event that triggers the onset of GDM based on the higher acylcarnitine concentrations shown in this study in women who developed GDM and can be used as biomarkers for early identification of women who will develop GDM.

Studies focused on the metabolic changes post-partum of women diagnosed with GDM have identified that lipid and amino acid metabolism dysregulation is strongly associated with the development of other metabolic diseases; these metabolic changes predispose women to undergo GDM in further pregnancies [57–59]. However, to date, no study has evaluated the progression to GDM in those women who did not undergo this pathology in previous pregnancies but further developed GDM in future pregnancies. Interestingly, not only these identified biomarkers could be used for the assessment of women who will undergo GDM during their current pregnancy; but they could certainly allow for the identification of those women who are at risk of developing this pathology in further pregnancies, even though they have not been diagnosed with GDM; this might be the case of the control women assessed in this study, whom the prediction model misclassified as part of the GDM group but did not develop this pathology. This study hypothesizes that a subclinical GDM-like condition is already present in these women, characterized by an impairment of lipid and/or amino acid metabolism that could probably trigger the development of GDM in future pregnancies. Further studies targeted to identify the metabolomics profile of healthy women who later develop GDM in further pregnancies are needed to elucidate this metabolic shift.

The present study has several strengths. First, to this research´s knowledge, it is the first study to evaluate and assess the fasting metabolomic profile of Mexican women who later develop GDM. This approach allows us to control the metabolic variation caused by postprandial states to determine amino acid and acylcarnitines concentrations. This investigation also controlled the model's validity by adjusting covariables such as maternal age, parity, and BMI, demonstrating that metabolomic variables alone can identify women who will develop GDM at weeks 24 to 28 of gestation. Second, compared to other models that included only clinical variables, reaching an AUC average of 0.70 (95% CI), the model generated in this study using only metabolomic variables can reach an AUC average of 0.91 (95% CI). There are, however, some limitations to this discovery study. First, the small sample size may restrict the inclusion of other metabolites into the model and improve the current model's specificity. A validation phase of these biomarkers in different populations is needed to evaluate GDM's new early diagnostic test. Replicating findings may improve understanding of GDM pathogenesis and may have implications for developing GDM prevention and early diagnosis protocols.

The benefits of early screening for GDM based on various risk profiles and/or metabolomics settings on maternal and infant outcomes have yet to be adequately evaluated by randomized controlled trials. Evidence regarding this issue is limited and non-conclusive; however, meta-analyses published in 2016 [60] and 2021 [61]; as well as the major guidelines for screening and treatment of GD [62, 63]; strongly encourage strict glucose monitoring and control in order to prevent short and long-term maternal and fetal outcomes. One of the main areas where metabolomics has already demonstrated significant potential is the discovery of biomarkers. Therefore, efforts should be made to target translational approaches in clinical practice to improve diagnosis and prevent adverse outcomes [64]. Further studies are needed to validate the applicability and cost-effective translational interventions of metabolomics on GDM, strategies that escape the scope of this study.

## Conclusions

This study demonstrates that early pregnancy maternal metabolites can be used to screen and identify pregnant women who will later develop GDM. The proposed model showed a predictive performance of 0.93 (0.869–0.991, 95% CI). This work also supports the hypothesis that higher acylcarnitine levels may be related to incomplete fatty acid β-oxidation and BCAA metabolism impairment in early pregnant women who will later develop GDM. Nevertheless, these results must be taken cautiously due to the limited sample size.

## Supplementary Information

**Additional file 1: Figure S1**. Acylcarnitines concentrations in the early stages of pregnancy. Results are shown as mean (±S.D.)*p<0.05 Mann Whitney U test for independent samples.

**Additional file 2: Figure S2**. Amino acid concentrations in the early stages of pregnancy. Results are shown as mean (±S.D.)*p<0.05 Mann Whitney U test for independent samples.

## Data Availability

The data presented in this study are available in the article or Supplementary Material.
