# Supplementary figures and images for: An early prediction model for gestational diabetes mellitus based on metabolomic biomarkers
Source: Diabetol Metab Syndr. 2023 Jun 1;15:116. doi: 10.1186/s13098-023-01098-7 (PMC10234027; doi:10.1186/s13098-023-01098-7)

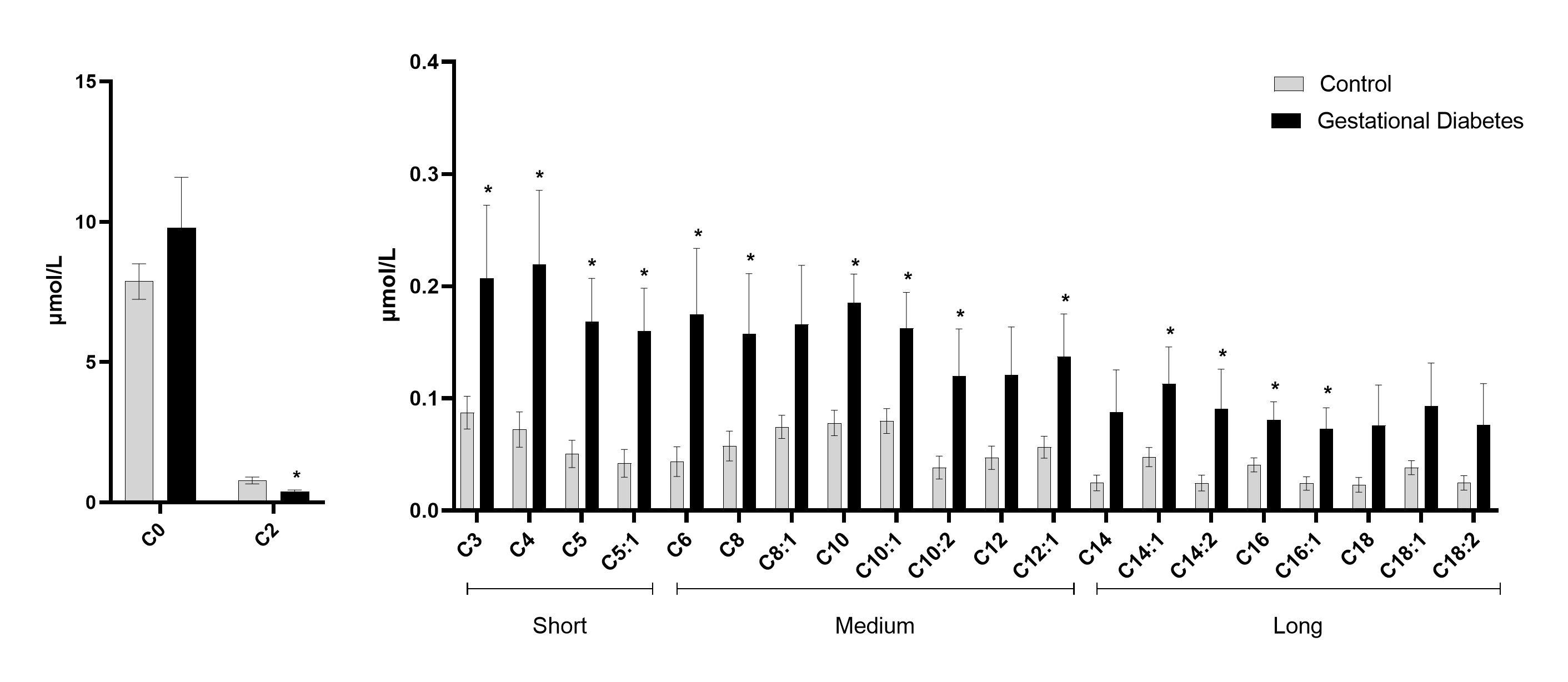

Supplement: Supplementary file 1 — Additional file 1: Figure S1. Acylcarnitines concentrations in the early stages of pregnancy. Results are shown as mean (±S.D.)*p<0.05 Mann Whitney U test for independent samples. [file 13098_2023_1098_MOESM1_ESM.tif]

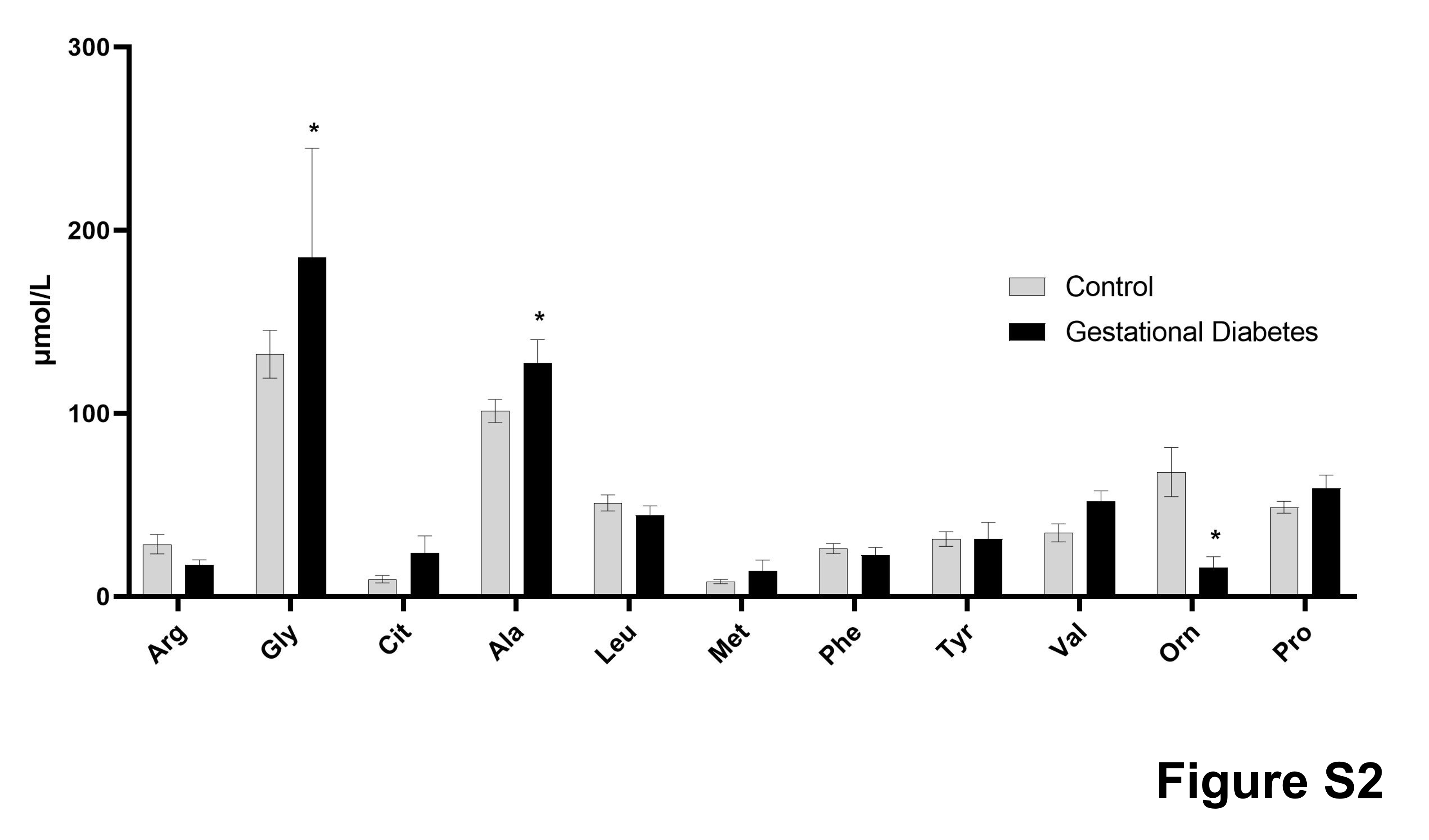

Supplement: Supplementary file 2 — Additional file 2: Figure S2. Amino acid concentrations in the early stages of pregnancy. Results are shown as mean (±S.D.)*p<0.05 Mann Whitney U test for independent samples. [file 13098_2023_1098_MOESM2_ESM.tif]
